# Oral administration of *Limosilactobacillus reuteri* VHProbi^®^ M07 alleviates ovalbumin-induced allergic asthma in mice

**DOI:** 10.1371/journal.pone.0317587

**Published:** 2025-01-16

**Authors:** Guoqing Meng, Hongchang Cui, Congrui Feng, Chaoqun Guo, Lei Song, Zhi Duan

**Affiliations:** 1 College of Agriculture and Bioengineering, Heze University, Heze, China; 2 College of Food Science and Engineering, Ocean University of China, Qingdao, China; 3 Qingdao Vland Biotech Group Co., Ltd, Qingdao, China; King Abdulaziz University Faculty of Medicine, SAUDI ARABIA

## Abstract

**Aims:**

Asthma is characterized by chronic airway inflammation, persistent cough, wheezing, and dyspnea. This study aimed to evaluate the efficacy of *Limosilactobacillus reuteri* VHProbi^®^ M07 (M07) administration in alleviate the asthma severity in a mice model.

**Methods and results:**

*In vitro* studies confirmed that M07 can survive and proliferate within the gastrointestinal tract. BALB/c mice were administered M07 both before and after ovalbumin (OVA) challenge. Serum levels of OVA-specific immunoglobulin (Ig) E and IgG1, inflammatory cells and cytokines in bronchoalveolar lavage fluid were assessed, along with histopathological examination of lung tissue. Compared to the placebo (PLA) group, mice treated with M07 exhibited significantly lower levels of OVA-specific IgE and IgG1 (*P* < 0.01). The counts of eosinophils and neutrophils were also significantly reduced in both the pretreated (PRE) group and post-treated (POS) group compared with the PLA group (*P* < 0.01). Histological analysis of lung tissues verified the protective effects of M07 against inflammation, demonstrating reduced infiltration of inflammatory cells. Additionally, mice in the PRE and POS groups showed significantly increased levels of IL-10 (*P* < 0.01), and significantly decreased levels of IL-5, IL-13, MCP-1, eotaxin, and tumor necrosis factor-α (*P* < 0.01).

**Conclusions:**

Oral administration of M07 mitigated key features of inflammatory responses in the OVA-induced mice asthma model. These findings suggest that M07 holds therapeutic potential for the treatment of allergic asthma.

## Introduction

Asthma is characterised by recurrent airway limitations and respiratory symptoms such as wheezing, coughing, breathlessness, and chest tightness. It is one of the most common chronic, non-communicable diseases globally [1]. Despite its relatively low mortality rate, asthma imposes a significant burden on healthcare systems and adversely affects patients and their families [2, 3]. Estimates suggest that over 334 million individuals worldwide are afflicted by asthma, with projections this number may rise to 400 million by 2025 [4].

The prevalence of asthma is highest in developed countries, where it has stabilized or is decreasing, and lowest in many developing countries, where it is increasing rapidly due to modernized lifestyles [1, 5]. Generally, chronic inflammation, airway obstruction, and hyperresponsiveness are the primary mechanisms involved in the pathogenesis of asthma [6]. Specifically, chronic airway inflammation is a hallmark feature of asthma regardless of disease duration and severity [7].

From an immunological perspective, three main processes are involved in the development of asthma: sensitization, challenge, and inflammation [8–11]. Elevated levels of antigen-specific immunoglobulins and proinflammatory cytokines, such as interleukin (IL)-4, IL-9 and IL-13, are observed in the serum accompanied by eosinophil infiltration as evidenced by histological findings [12–15]. Commonly, pharmacological treatments, including inhaled corticosteroids (ICS), short-acting and long-acting β_2_ agonists (SABA/LABA) are recommended for asthma management [16–18]. However, given the limitations of these therapies—such as poor adherence, over-reliance, and an increased risk of exacerbation and mortality—developing new treatments that target the underlying immunological pathways to enhance efficacy and improve asthma control is a rational approach [19–22].

In recent decades, the potential impact of intestinal microbiota dysbiosis on the pathogenesis of respiratory illnesses and associated disease symptoms has garnered increasing attention [23–25]. The maturation of host’s immune system and its susceptibility to diseases such as respiratory infections and asthma are closely linked to the composition and diversity of gut microbiota [26]. Consequently, restoring gastrointestinal microflora to promote host health through nonpharmacological interventions, including probiotics, represents a promising approach.

Probiotics have been defined as live microorganisms that, when administered in adequate amounts, confer a health benefit on the host [27]. Studies have suggested that probiotics can be utilized to prevent and/or alleviate asthma due to their anti-inflammatory and immunomodulatory properties [28]. For instance, Zhang et al. demonstrated that oral administration of *Clostridium butyrium* can prevent and treat airway inflammation in mice with ovalbumin (OVA)-induced asthma by exerting immunoregulatory effects [29]. Drago et al. conducted a clinical trial using a mixture of probiotics to ameliorate asthma severity, showing that certain probiotic strains can serve as adjuvant therapies [2]. Given the high degree of heterogeneity among probiotic strains, including their immunological characteristics and genetic backgrounds, identifying additional microorganisms with potential probiotic properties to enhance efficacy is of significant importance [30].

Previously, we isolated and characterized the probiotic strain *Limosilactobacillus reuteri* VHProbi^®^ M07 (hereafter referred as to “M07”) based on its phenotypic and genotypic properties [31]. In this study, we investigated whether M07 could prevent and/or mitigate asthma using an OVA-induced asthma model in mice.

## Methods and materials

### Culture conditions

M07 was inoculated in the de Man-Rogosa-Sharpe (MRS) broth medium (Beijing Land Bridge, China) and incubated anaerobically overnight at 37°C. This experiment was conducted from May 1, 2020 to May 30 2020 and was approved by the Ethics Committee of Qingdao Vland Biotech Inc. with approval number VL-HC-20200329. Fermented cultures with a live bacterial count of 1×10^^9^ CFU/mL were directly used for subsequent experiments [32].

### Tolerance of M07 to simulated gastrointestinal (GI) juice

The protocol was developed based on the method described by Millette [33]. Briefly, porcine pepsin (Sigma) and pancreatin (Sigma) were added to simulated gastric juice and intestinal fluid, respectively, and incubated in a water bath at 37°C for 1 h before use. To simulate gastrointestinal transit, 1 mL of fermented supernatants were mixed with 9 mL of simulated gastric juice and incubated for 2 h at 37°C. Subsequently, 1 mL of this mixture was co-incubated with 24 mL of simulated intestinal fluid for 3 h at 37°C. Enumeration of live bacteria was conducted before and after the simulated digestion to evaluate M07’s resistance to gastric acid and bile salts. The assay was repeated twice.

### Adhesion of M07 to Caco-2 cells

The adhesive property of M07 to the intestinal epithelial cells was examined according to the method described by Berenet [34]. Briefly, Caco-2 cells were seeded on glass coverslips and washed twice with phosphate-buffered saline (PBS, Sigma). Culture medium (1mL) was added to achieve a final concentration of 2 × 10^^5^ cells/well. 200 μL of bacterial supernatant (1 × 10^^9^ CFU/mL) were added to the 96-well plates, and the mixture was incubated in a CO_2_ incubator at 37°C for 2 h. The monolayers were then washed three times with PBS, fixed and stained for microscopic examination. Subsequently, the monolayers were co-incubated with trypsin, and the number of live bacteria was counted using the conventional plate count method. The test was performed in duplicate. The Adhesion Index (CFU/cell) was calculated as the total bacterial count per well divided by the total cell counts per well.

### Animal experiments

Twenty-four specific pathogen-free (SPF) BALB/c mice, aged 8–9 weeks, were obtained from the Pengyue Experimental Animal Centre (animal permit number SCXK(Lu)20140007; Jinan, Shandong). The mice were provided with standard pellet feed and water *ad libitum*. They were housed in an environment maintained at 20–26°C, with relatively humidity of 40–70%, and exposed to a 12-h light-dark cycle [35]. Housing conditions, ventilation, air quality, caging, bedding, and quarantine met the requirements of SPF mice. The animal experiment was conducted under the supervision and approval of the Laboratory Animal Ethic Committee of Nanjing BIOGENE Biotech Co., Ltd (approval number:20230710105501). The study adheres to the ARRIVE guidelines.

Mice were adaptively fed for 7 days. Subsequently, the mice were randomly divided into four groups (n = 6 per group): normal (NOR), placebo (PLA), pre-treated probiotics (PRE), and post-treated probiotics (POS). All mice were provided with a regular diet throughout the study period. Mice in the PRE group received 200 μL of M07 suspension (1 × 10^^9^ CFU/mL) by gavage once daily for 10 days prior to the first sensitization until the study ended. Mice in the POS group were administered the same volume of M07 suspension from day 0 to day 27. Mice in the NOR and PLA groups received 200 μL of sterile saline solution (0.85%) daily from day 0 to day 27.

**Fig 1:**
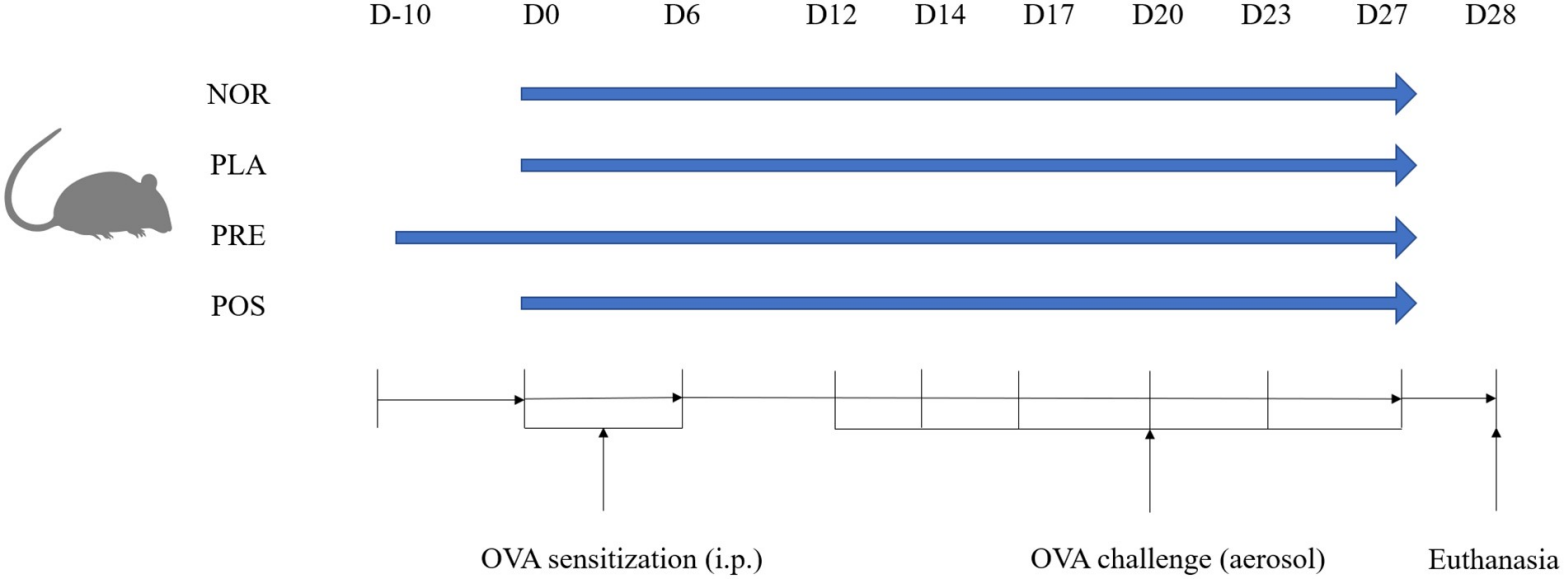
Schematic of the OVA-induced asthma mouse model protocol. NOR, normal group; PLA, placebo group; PRE, pre-treated group; POS, post-treated group; OVA, ovalbumin; i.p. intraperitoneal; Dn, Day n.

To establish an asthma model, mice in all groups except the NOR group were intraperitoneally injected with 200 μL of an allergen solution containing ovalbumin (OVA, 50 μg; Shanghai Yuanye Bio-Technology, Shanghai, China) and aluminium hydroxide (800 μg) dissolved in saline on days 0 and 6. The allergen solution was filtered and sterilized prior to use to ensure contamination-free conditions. Mice were also exposed to aerosolized OVA (2% in saline) using an air pressure atomizer (OMRON^®^ C28S) for 30 min on days 12, 14, 17, 20, 23, and 27 to exacerbate asthma symptoms. The dose of the allergen administered via aerosol was based on the duration of exposure. Meanwhile, mice in the NOR were treated with the same volume of PBS during sensitization and challenge. All mice were euthanized using pentobarbital sodium (2%, 0.045 mL/g body weight) on day 28. The asthma model was established as shown schematically in Fig 1, with slight modifications from the method described by Qian‑Lin Yu [36].

#### Detection of OVA-specific immunoglobulin (Ig)E and IgG1

Blood samples were collected from the orbital venous plexus of all mice after the mice immediately after euthanasia on day 28 and stored at 4°C overnight. The next day, blood samples were centrifuged at 3000 rpm for 15 min, and serum was obtained for OVA-specific IgE and IgG1 assays using enzyme- linked immunosorbent assay (ELISA) kits (Beyotime) according to the manufacturer’s instructions [37].

#### Determination of cytokines and inflammatory cells in bronchoalveolar lavage fluid

Tracheotomy was performed immediately after euthanasia. Bronchoalveolar lavage fluids (BALF) was obtained by flushing the lungs three times with 800 mL of PBS. The BALF samples were centrifuged at 1200 rpm for 10 min at 4°C. The pelleted cells were resuspended in 1 mL of PBS and stained with Trypan blue (Yuanye, CN) to count viable cells. Cell smears were prepared using a cytospin centrifuge (ELITechGroup), stained with methvlem blue and Eostm Y (Sigma), and examined under an Olympus^®^ microscope to differentiate cell types based on cytochemical and morphological characteristics. The supernatants were collected to determine cytokine levels using ELISA kits such as interleukin-5 (IL-5), IL-10, IL-13, monocyte chemoattractant protein-1 (MCP-1), tumor necrosis factor-alpha(TNF-α), and eotaxin [38].

#### Histology of lung tissue

Lung tissues were fixed in 10% formalin for 24h. Paraffin blocks were prepared, and sections (5μm thick) were obtained. The tissue sections were stained with hematoxylin and eosin (HE, Promega), and examined under a light microscope at×400 magnification (LEICA, Germany) [39].

### Statistical analysis

Data are presented as means ± standard deviations. Differences in the mean values among groups were analyzed using one-way analysis of variances (ANOVA) followed by Duncan’s multiple range test. *P* value < 0.05 and < 0.01 was defined as statistically significant and extremely significant differences, respectively. Statistical analysis was conducted using SPSS 20.0 (SPSS Inc, USA). GraphPad Prism 8 (GraphPad Software Inc, USA) was used to generate plots.

## Results

### Tolerance to artificial GI juice

*In vitro* studies demonstrated that M07 exhibits excellent tolerance to simulated GI fluid. The enumeration of live bacteria is shown in Table 1. The initial count was 9.86 Log CFU/mL, which decreased to 9.33 Log CFU/mL after co-incubation with simulated gastric juice. The final surviving bacterial count was 9.02 Log CFU/mL, representing a reduction of 0.84 Log compared to the initial count. Therefore, M07 has the potential to survive in the gastrointestinal tract.

**Table 1:** Viability of M07 after co-incubation with GI fluid (Log CFU/mL).

	Initial count	After 2 h in simulated gastro juice	After 3 h in simulated intestinal fluid
Live bacteria count	9.86 ± 0.27	9.33 ± 0.32	9.02 ± 0.18

Values are presented in mean ± standard deviation.

### Adherence to the Caco-2 cells

**Fig 2:**
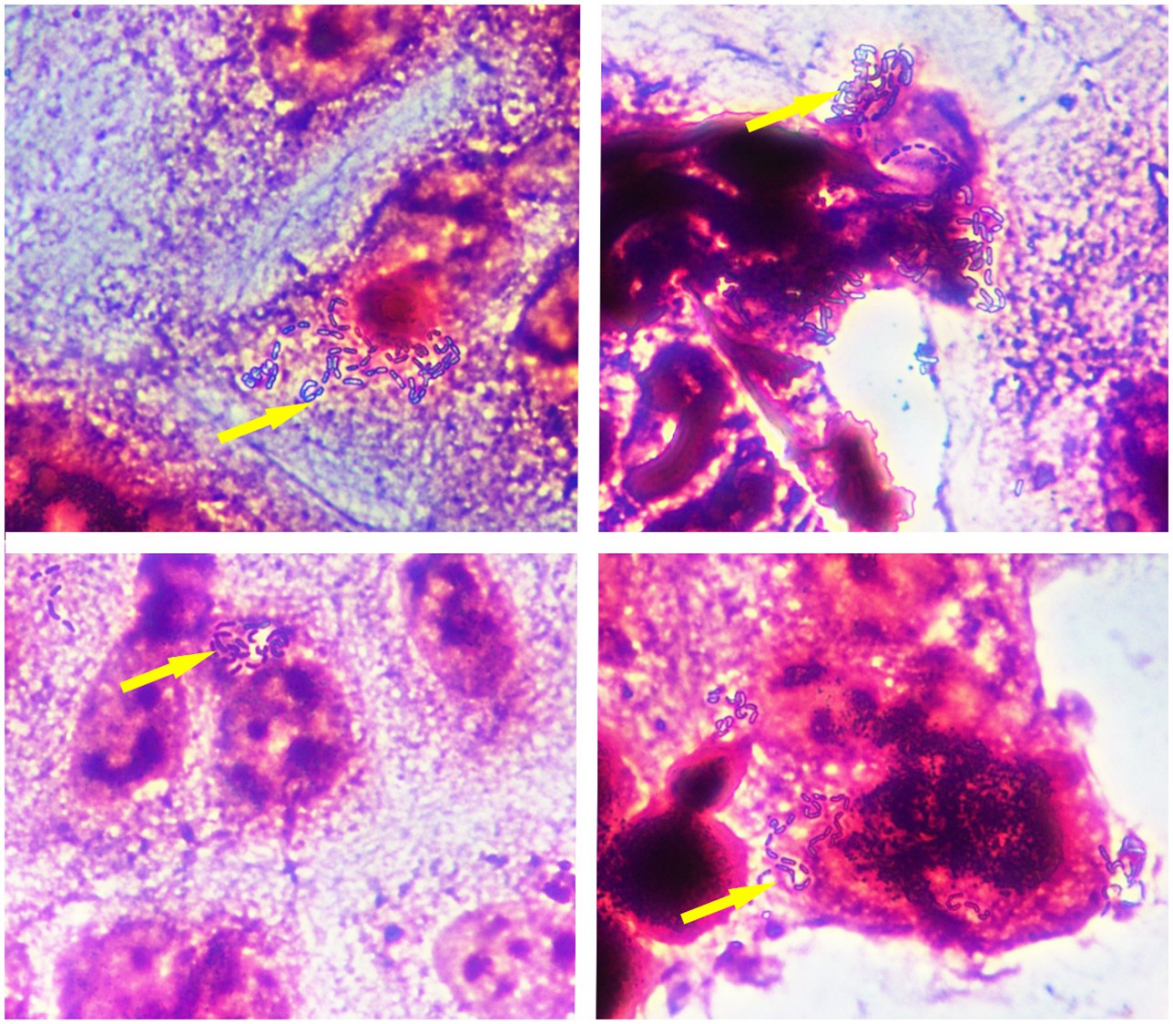
Microscopic examination of M07 adhesion to Caco-2 cells (400x). Yellow arrows indicate bacterial cells adhered to Caco-2 cell after washing.

Microscopy results showed that M07 was capable of adhering to the surface of Caco-2 cells (Fig 2). The Adhesion Index value was 23.18 ± 1.52. This indicates that M07 has the potential to colonize human intestinal epithelial cells.

### Levels of OVA-specific IgE and IgG1 in serum

**Fig 3:**
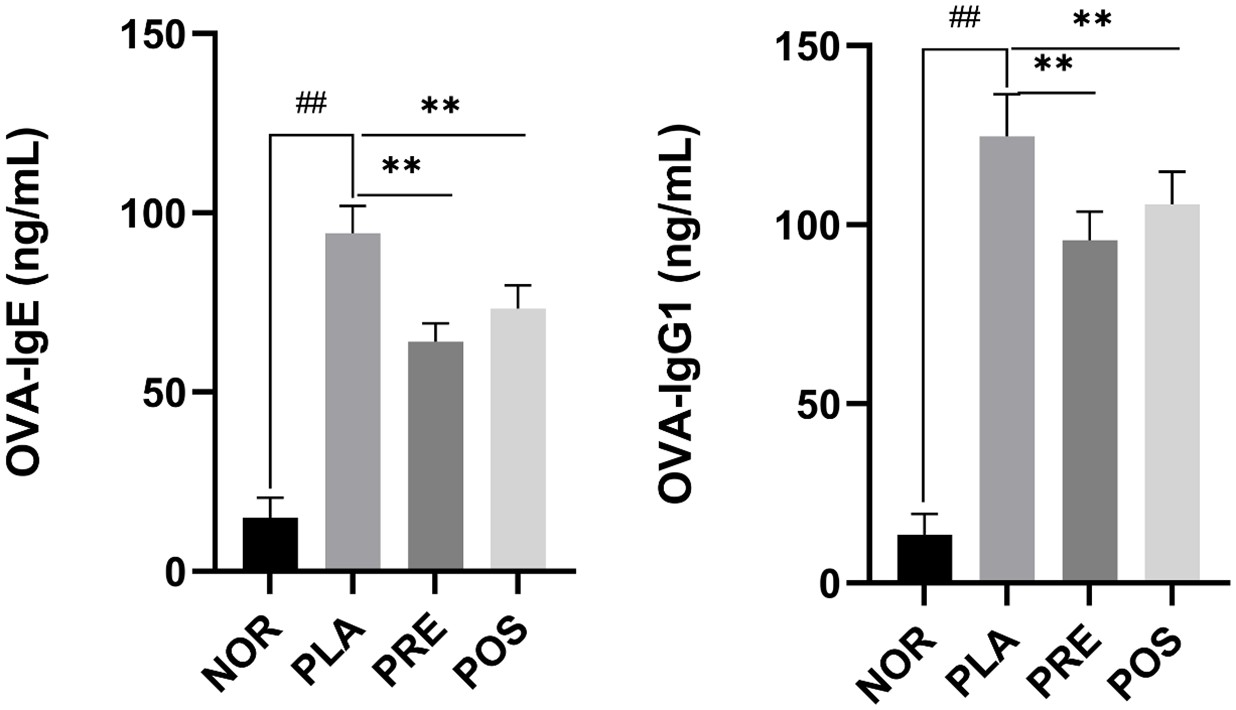
Levels of OVA-specific IgE and IgG1 in serum from different groups of mice. NOR, normal group; PLA, placebo group; PRE, pre-treated group; and POS, post-treated group. OVA, ovalbumin; IgE, immunoglobulin E; IgG1, immunoglobulin G1; ^##^*P* < 0.01 compared with NOR; **P < 0.01 compared with PLA.

Serum was collected to determine the levels of OVA-specific IgE and IgG1. Compared with the NOR group, the levels of OVA-specific IgE and IgG1 were significantly higher in the PLA group (*P* < 0.01) (Fig 3), indicating that OVA-sensitized mice underwent allergic reactions. In the PRE group, the level of OVA-specific IgE decreased by 32.0% (*P* < 0.01) and the level of OVA-specific IgG1 decreased by 23.2% (*P* < 0.01) compared with the PLA group. The levels of OVA-specific IgE and IgG1 in the POS group were also significantly lower than those in the PLA group (*P* < 0.01), but higher than those in the PRE group.

### Enumeration of differential leukocytes in BALF

The total numbers of inflammatory cells and eosinophils in the BALF were significantly increased in the PLA group compared with those in the NOR group (*P* < 0.01) (Table 2). The lymphocyte count in the PLA group was 4.67-fold higher than that in the NOR group, which was significantly different (*P* < 0.01). These data indicate that the OVA-induced asthma mouse model has been successfully established.

**Table 2:** Differential counts of leukocytes in mouse BALF.

Group	Total cell	Eosinophils	Lymphocyte	Macrophage
	*10^^4^	*10^^4^	*10^^4^	*10^^4^
NOR	3.60 ± 1.27^a^	0.36 ± 0.20^a^	4.48 ± 2.95^a^	1.33 ± 0.54^a^
PLA	44.90 ± 10.02^b^	16.51 ± 2.65^b^	25.40 ± 6.84^b^	2.56 ± 1.31^b^
PRE	18.70 ± 3.12^b^^c^	3.36 ± 1.14^a^	18.81 ± 6.55^b^	1.89 ± 0.76^a^^b^
POS	25.73 ± 8.56^b^^c^	5.38 ± 1.52^b^^c^	20.69 ± 6.28^b^	2.28 ± 0.69^a^^b^

NOR, normal group; PLA, placebo group; PRE, pre-treated group; and POS, post-treated group. Different letters indicate statistically significant differences among groups (*P*< 0.01).

^a^, compared with NOR;

^b^, compared with PLA;

^c^, compared with PRE.

Mice in the PRE and POS groups that received M07 gavage showed a significant reduction in total cell numbers and eosinophil count compared with mice in the PLA group (*P* < 0.01). Specifically, the total cell counts in the PRE and POS groups decreased by 58.4% and 42.7%, respectively, compared with the PLA group. The eosinophil counts in the PRE and POS groups decreased by 79.6% and 67.4%, respectively, compared with PLA group. There were no significant differences in lymphocyte or macrophage counts among the four groups.

### Concentration of different cytokines in BALF

**Fig 4:**
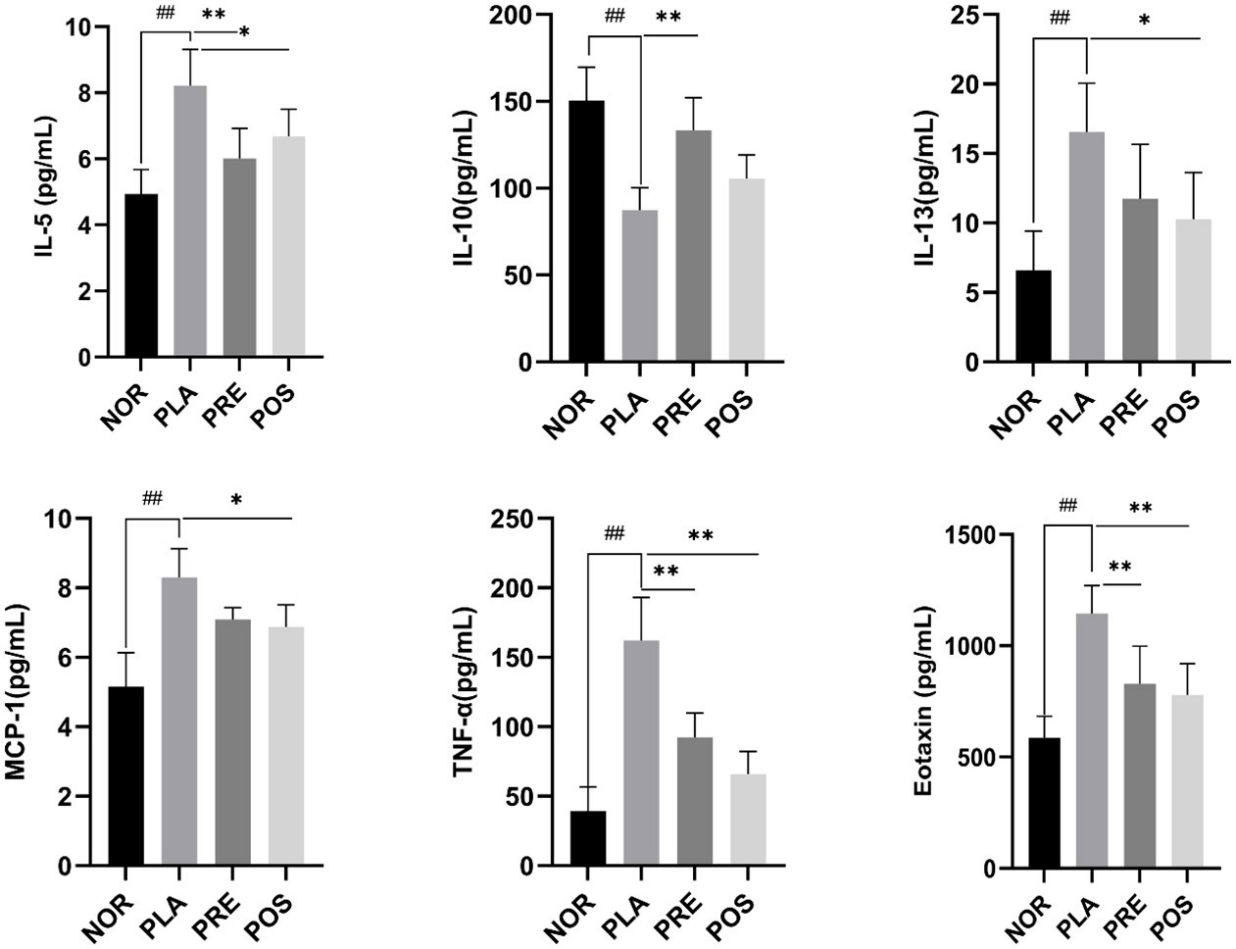
Levels of pro- and anti-inflammatory cytokines in BALF. NOR, normal group; PLA, placebo group; PRE, pre-treated group; and POS, post-treated group. IL, interleukin; MCP-1, monocyte chemotactic protein-1; TNF-α, tumor necrosis factor- alpha. ^##^*P* < 0.01 compared with NOR; * *P* <0.05, ** *P* <0.01.

Detailed changes in cytokine levels are depicted in Fig 4. After establishing the asthma model, the levels of IL-5, IL-13, MCP-1, TNF-α, and eotaxin significantly increased in the PLA group compared with those in the NOR group (*P* < 0.01), while the IL-10 level significantly decreased by 42.0% (*P* < 0.01). Mice that underwent gavage with M07 in the PRE and POS groups showed a significantly lower level of IL-5 compared with the PLA group (*P* < 0.05); specifically, the IL-5 level decreased by 26.8% in the PRE group and 18.7% in the POS group.

The level of the anti-inflammatory cytokine IL-10 in the PRE group was significantly higher than that in the PLA group (*P* < 0.01), increasing by 20.8%. There was no significant difference in IL-10 levels between the PLA and POS groups (*P* > 0.05). The levels of IL-13 and MCP-1 in the POS group were significantly lower than those in the PLA group (*P* < 0.05), decreasing by 38.0% and 17.2%, respectively. The TNF-α level in both the PRE and POS groups was significantly lower than that in the PLA group (*P* < 0.01), reducing by 43.0% and 59.4%, respectively.

Meanwhile, the level of eotaxin exhibited a similar trend to that of IL-5, decreasing by 27.6% in the PRE group and 31.9% in the POS group, with significant differences compared with the PLA group (*P* < 0.01).

### Histopathological examination of the lung tissue

**Fig 5:**
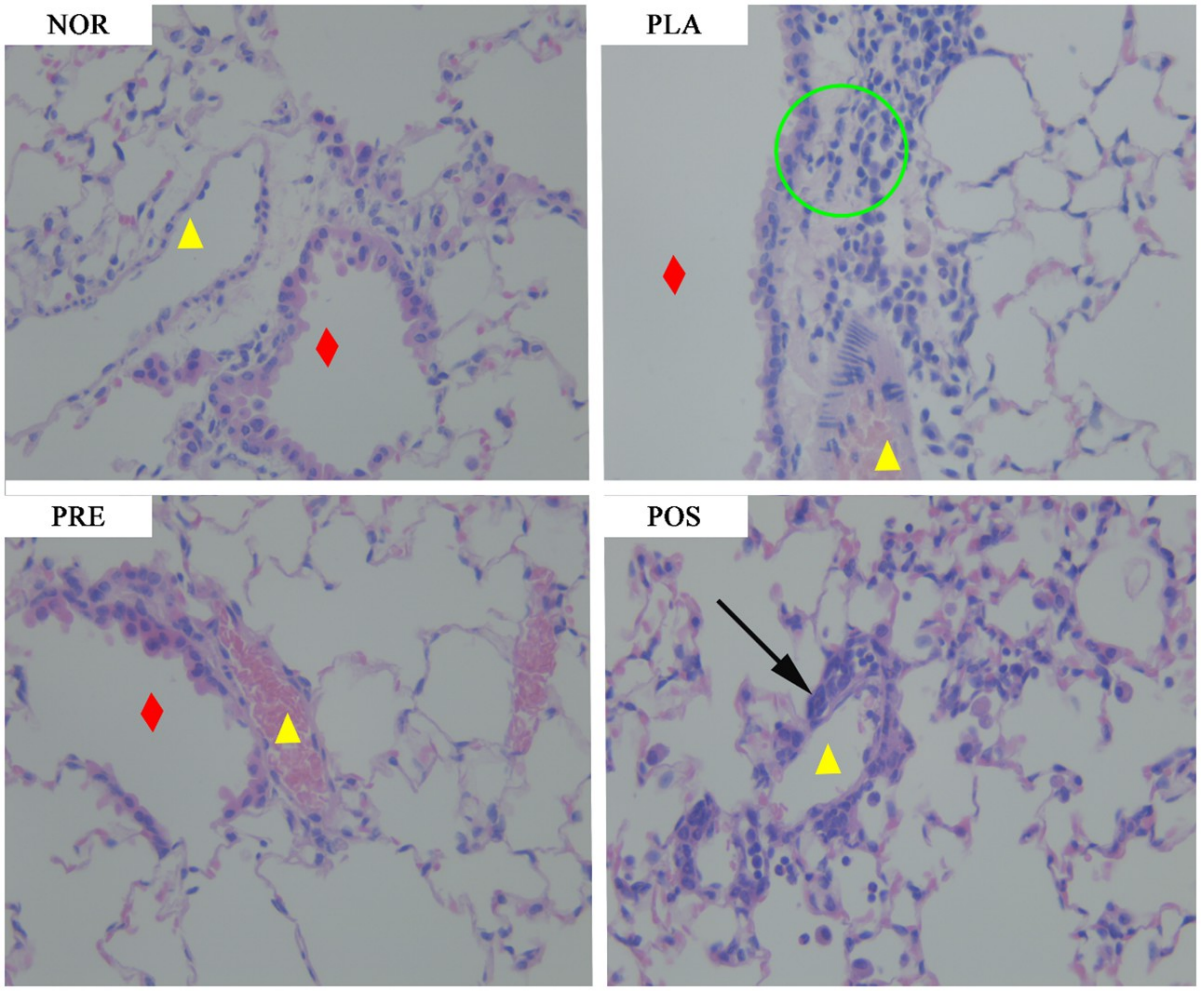
Representative sections of HE-stained lung tissue from different groups under light microscopy(400X). NOR, normal group; PLA, placebo group; PRE, pre-treated group; and POS, post-treated group. The yellow triangle indicates vessels; the red rhombus indicates bronchioles; the black arrow indicates infiltration of inflammatory cells; the green circle indicates inflammatory hyperplasia.

The lung tissues from each mouse were dissected and stained with HE. Changes in the structure of pulmonary alveoli, bronchi, and the infiltration of inflammatory cells were qualitatively analyzed. Mice in the NOR group exhibited normal respiratory epithelium within branching bronchioles (Fig 5). Pulmonary alveoli primarily consisted of type *I* and type *II* alveolar cells. There was no infiltration of inflammatory cells into peribranchial, perivascular, or intra-alveolar spaces.

Mice in the PLA group showed inflammatory hyperplasia around terminal bronchioles, which extended into larger bronchi. The number of macrophages in the pulmonary alveoli increased significantly. In the PRE group, mice had an increased number of macrophages in the pulmonary alveoli but no other significant inflammatory changes. Mice in the POS group had vasodilated vessels in lung tissues, along with significant infiltration of inflammatory cells and an increased number of macrophages.

## Discussion

In recent decades, various studies have demonstrated the existence of bidirectional communication between the respiratory system and gastrointestinal tract, known as the gut-lung axis [26, 40, 41]. Dysbiosis of gut commensal microflora appears to play a crucial role in the initiation and progression of allergic diseases. For instance, germ-free mice tend to develop immune system disorders when exposed to allergens due to the absence of gut microbiota [42, 43]. In recent years, a prevailing hypothesis has emerged, suggesting that allergic disease, including asthma, atopic dermatitis, and rheumatoid arthritis, are closely related to lifestyle factors that influence the diversity and abundance of microbiome, such as diet, antibiotic use, and hygiene practices [44–47].

Manipulating the human gut microflora to enhance immune functions, particularly through probiotic supplementation, has increasingly drawn global attention [48–50]. For example, Moura et al. conducted a small clinical study and demonstrated that supplementation with *L*. *reuteri* as an adjuvant therapy could mitigate the severity of asthma in children and adults [51]. Forsythe et al. showed that oral administration of *L*. *reuteri* improved the major characteristics of OVA- sensitized asthma in mice, including cytokine levels, eosinophilia, and airway hyperresponsiveness [52]. Raftis et al. found that *Bifidobacterium breve* MRx0004 could protect against inflammatory cell infiltration in a severe asthma model by reducing neutrophil and eosinophil counts [38]. Therefore, developing non-pharmacological strategies, such as novel probiotic strains for the clinical management of asthma, brings substantial benefits to patients and society.

The gastric juice and intestinal fluids pose a primary challenge for probiotics to survive in the harsh gut environment [53]. Probiotics with favourable tolerance to GI fluids are more likely to colonize in the gut tract, thereby conferring beneficial effects on the host. We demonstrated that the loss of M07 was less than 1 Log cycle after successive co-incubation with simulated gastric and intestinal juice, indicating its robust survival capability. The ability to hostile conditions is a fundamental characteristic of effective probiotic dietary supplements. Adherence to the epithelial cells is a prerequisite for probiotics to confer benefits in the GI tract, including competing for binding sites and nutrients with harmful gut microbiota and communicating with the host immune system [54–56].

Caco-2 cell lines derived from human colon adenocarcinoma are commonly used as an *in vitro* model to assess the adhesion properties of probiotics [57]. Our results showed that M07 could adhere to Caco-2 cells, as evidenced by microscopic examination and cell counts. This finding aligns with studies on extensively researched probiotics such as *Lacticaseibacillus rhamnosus* GG, which exhibits strong affinity for intestinal mucosal cells and resistance to gastric acids and bile salts [58]. Similarly, *Lactobacillus acidophilus* NCFM has demonstrated the capacity to adhere to Caco-2 cells and resist gastrointestinal fluids [59, 60]. These consistent findings suggest that M07 has the potential to colonize in the human gut tract.

From the perspective of pulmonary immunology, asthma has been considered a hallmark of T-help type 2 (Th2) disorders in the airways [12]. Elevated levels of IgE and IgG1 are indicative of a Th2-type adaptive immune response [61]. Mice in the PLA group exhibited significantly increased levels of OVA-specific IgE and IgG1 in serum, indicating a Th2 immune response. Mice treated with M07 displayed significantly reduced levels of OVA-specific IgE and IgG1 compared with those in the PLA group. Similar results have also been observed in various OVA-induced mice model experiments.

For example, Hougee et al. conducted a comparative study and demonstrated that oral treatment with *B*. *breve* M-16V or *Lactiplantibacillus plantarum* NumRes8 reduced the levels of OVA-specific IgE and IgG1 in OVA-sensitized mice [61]. Lan and colleagues investigated the efficacy of *L*. *plantarum* CQPC11 in attenuating airway inflammation in OVA-induced mice and documented reduced serum levels of OVA-specific IgE and OVA-specific IgG1 [62]. Furthermore, Hagner et al. illustrated that intranasal administration of *Staphylococcus sciuri* W620 in an OVA-exposed mouse model led to significantly reduced serum levels of OVA-IgG1 and IgE compared with untreated mice [63]. Taken together, these data suggest that consumption of M07 could relieve the severity of asthma by suppressing serum levels of both OVA-specific IgE and IgG1.

Airway inflammation is a pathogenic factor in asthma and involves leukocytes such as eosinophils, neutrophils, lymphocytes, and macrophages [64, 65]. Restoring the dysfunction of immune cells plays a crucial role in alleviating allergic diseases and associated inflammation [66]. In mice that received M07 gavage, the counts of total inflammatory cells and eosinophils were significantly reduced compared with those in the PLA group and approached levels seen in the NOR group. These reductions were consistent with declines in eotaxin levels. There were no significant differences in the counts of lymphocytes and macrophages among mice in PLA, PRE and POR groups.

Raftis et al. discovered that severe asthma mice treated with *B*.*breve* MRx0004 showed therapeutic effects by suppressing neutrophil and eosinophil counts in lung tissues [38]. Forsythe and coworkers demonstrated that oral treatment with live *L*. *reuteri* ATCC 23272 could assist in treating allergic airway responses in mice by reducing eosinophil infiltration into the airway lumen and parenchyma [52]. Spacova et al. found that intranasal administration of *L*. *rhamnosus* could prevent birch pollen-induced allergic asthma in a murine model, with significantly lower eosinophil counts in BALF [67].

Our results are consistent with these studies, which describe reductions in pulmonary eosinophil numbers in asthma mouse model using probiotics. Oral administration of M07 could attenuate asthma severity by suppressing the number of total leukocytes and eosinophils.

IL-5 and IL-13 are pivotal in the development of an allergen-sensitized asthma model. IL-5 is responsible for the maturation and differentiation of eosinophils [1]. Delivering IL-13 to the airways induces asthma symptoms such as eosinophil infiltration and a vigorous response in the airway, while inhibition of IL-13 levels could attenuate these effects [11, 68]. IL-10 is an immunosuppressive cytokine, and clinical studies have revealed that serum IL-10 level can reflect the severity of asthma [69].

Analysis of cytokine levels in BALF revealed elevated levels of pro-inflammatory cytokines (IL-5, IL-13, MCP-1, and TNF-α) in the PLA group, while a decreased level of the anti-inflammatory cytokine of IL-10 was observed. Mice treated with M07 via gavage displayed diametrically opposite trends in cytokine levels compared with those in the PLA group. Mice pretreated with M07 in the PRE group showed greater recovery of cytokine levels compared with those in the POS group, although no significant difference was observed between these two groups.

Zhang et al. found that administration of *L*. *rhamnosus* GG led to reductions in IL-5, IL-13, and MCP-1 levels and an increase in IL-10 levels in OVA-sensitized mice [70]. Oral administration of *L*. *reuteri* ATCC 23272 also resulted in immunoregulatory effects, with elevated IL-10 levels and reduced levels of IL-5, IL-13, MCP-1, and TNF-α in OVA-induced asthma mice [52]. One could speculate that oral administration of M07 suppress asthma development by regulating pro-inflammation and anti-inflammation cytokines.

M07 also confers enhanced effects on lung histopathology, as illustrated by the significantly reduced infiltration of inflammatory cells into perivascular and peribronchiolar areas. The improvements in lung tissue pathology were associated with prolonged M07 administration. The reduced infiltration of inflammatory cells was consistent with the decreased counts of eosinophils and neutrophils.

We acknowledge that the present study has certain limitations. Although M07 confer benefits in relieving OVA-induced asthma in mice, the mechanisms of action of M07 need to be investigated in greater detail. For instance, further research is required to understand how the airway responsiveness changes during asthma development. Additionally, the effect of M07 on the abundance and diversity of gut microflora should be explored. Moreover, it is crucial to accumulate positive evidence of the beneficial effects of M07 by conducting human clinical trials.

In conclusion, this study clearly showed that M07 can tolerate and survive in the harsh gastrointestinal environment. More importantly, it alleviates OVA-induced asthma in mice by reducing the Th2-type immune response, including decreased infiltration of inflammatory cells and specific antibody levels, as well as regulating of pro- and anti-inflammatory cytokines, thereby restoring immunological balance. Moreover, the efficacy of M07 in ameliorating asthma symptoms was greater in the PRE group compared to the POS group. Taken together, our findings provide a promising probiotic strain, M07, for managing allergic respiratory diseases and support the concept that oral administration of probiotics can benefit distal sites such as respiratory tract.

## Supporting information

S1 Data (XLSX)
